# Current advances in biomaterials for inner ear cell regeneration

**DOI:** 10.3389/fnins.2023.1334162

**Published:** 2024-01-12

**Authors:** Junze Lu, Man Wang, Yu Meng, Weibin An, Xue Wang, Gaoying Sun, Haibo Wang, Wenwen Liu

**Affiliations:** ^1^Department of Otolaryngology-Head and Neck Surgery, Shandong Provincial ENT Hospital, Shandong University, Jinan, China; ^2^Shandong Institute of Otorhinolaryngology, Jinan, China

**Keywords:** biomaterials, inner ear cell regeneration, hearing loss, hydrogel, conductive materials

## Abstract

Inner ear cell regeneration from stem/progenitor cells provides potential therapeutic strategies for the restoration of sensorineural hearing loss (SNHL), however, the efficiency of regeneration is low and the functions of differentiated cells are not yet mature. Biomaterials have been used in inner ear cell regeneration to construct a more physiologically relevant 3D culture system which mimics the stem cell microenvironment and facilitates cellular interactions. Currently, these biomaterials include hydrogel, conductive materials, magneto-responsive materials, photo-responsive materials, etc. We analyzed the characteristics and described the advantages and limitations of these materials. Furthermore, we reviewed the mechanisms by which biomaterials with different physicochemical properties act on the inner ear cell regeneration and depicted the current status of the material selection based on their characteristics to achieve the reconstruction of the auditory circuits. The application of biomaterials in inner ear cell regeneration offers promising opportunities for the reconstruction of the auditory circuits and the restoration of hearing, yet biomaterials should be strategically explored and combined according to the obstacles to be solved in the inner ear cell regeneration research.

## 1 Introduction

The inner ear is important in the management of hearing and balance. The cochlea, an intricate organ of hearing, is a delicate membranous labyrinth surrounded by dense temporal bone. Anatomically, the cochlea contains at least four functional domains including sensory epithelium, neuronal compartment, lateral wall, and immune cells (Milon et al., 2021). Among these compartments, hair cells (HCs) located on the sensory epithelium are the first-level receptors for auditory conduction, responsible for converting acoustic signals into electrical signals and transmitting acoustic information to the neuronal compartment (Sun et al., 2018). Spiral ganglion neurons (SGNs) located in the neuronal compartment receive the acoustic signals and transmit them upward to the central auditory system (Shrestha et al., 2018). The lateral wall consists of the stria vascularis and spiral ligament. They maintain blood flow to the cochlea and generate the endocochlear potential necessary for sensory HC transduction by secreting potassium ions into the endolymph (Zhang Y. et al., 2023). Immune cells maintain the stability of the inner ear environment and respond to inflammation in the inner ear (Zhang D.-G. et al., 2023). Multiple functional domains and various cell types in the cochlea interact to sustain hearing formation. These intricate and delicate structures make the cochlea a challenging target for both basic research and therapeutic intervention.

Hearing loss is a common sensory disorder in people and has a significant impact on reducing quality of life (Chadha et al., 2021). Sensorineural hearing loss (SNHL), one of the most common types of hearing loss, is caused by damage to inner ear HCs and SGNs (Wong and Ryan, 2015; Wang and Puel, 2018). HCs and SGNs in the inner ear are the core components involved in auditory information transfer (Coate and Kelley, 2013) and are susceptible to various external stimuli, such as noise (Chen et al., 2022), aging (Garg et al., 2021), ototoxic drugs (Wang et al., 2019), etc. Damage to mammalian HCs and SGNs could lead to permanent hearing loss, as they are terminally differentiated cells and cannot be self-regenerated (Daudet et al., 1998; Géléoc and Holt, 2014). Cochlear implantation (CI) helps to restore hearing in most patients with severe SNHL caused by HC damage (Naples and Ruckenstein, 2020). Nevertheless, the degeneration of SGNs may have a poor effect on CI (Webster and Webster, 1981) and there is currently no effective treatment for SNHL caused by SGN damage at present (Wang et al., 2022). Over the past few decades, important discoveries, including pharmacological therapies, genetic cell-based therapies, and biotherapies have led to the development of promising treatments for SNHL. However, there have been no approved medications or clinical therapies capable of rescuing or regenerating damaged HCs or SGNs.

Currently, stem/progenitor cell-based inner ear cell regeneration including inner ear organoids *in vitro* (Pouraghaei et al., 2020; Hocevar et al., 2021), elimination of the electrode-neuron gap after CI (Mattotti et al., 2015; Cai et al., 2016; Nella et al., 2022), and attempts at inner ear regeneration *in vivo* (Zhong et al., 2016; Chang H. T. et al., 2020) are some of the primary directions for inner ear regeneration research. Among these studies, the application of biomaterials and developments in bioengineering have also provided strategies to overcome the obstacles in inner ear cell regeneration (Brant et al., 2021). Biomaterials are defined as any natural or synthetic substance or a combination of substances that can interact with biological systems and can be used to improve biological function or life quality at any point of time (Marin et al., 2020). In recent years, biomaterial applications in the field of regeneration have been greatly developed owing to their excellent chemical versatility and biocompatibility (Bao et al., 2018; Liu et al., 2018). Biophysical signals transmitted by biomaterials, such as substrate stiffness, topography, mechanical forces and electric stimuli, have been proven to influence cell activity as well as cell fate determination (Shao et al., 2015; Yue et al., 2015; Ravikumar et al., 2017). For instance, some topical gels have been assessed to promote tissue regeneration and enhance diabetic foot ulcers (DFUs) wound healing in patients with diabetes (Bardill et al., 2022). Furthermore, Regranex gel has been approved by the Food and Drug Administration (FDA) as a growth factor mixture for clinical treatment of DFUs (Piascik, 1996). Biomaterials have also contributed significantly to the rapid advancement of both bone (Lee et al., 2018) and skin tissue regeneration technologies (Wu, 2021). Developments in bioengineering have also provided strategies to overcome obstacles in the process of inner ear cell regeneration (Brant et al., 2021). Several studies have reported that biomaterials play important roles in establishing inner ear organoids, promoting HCs and SGNs survival and regeneration, as well as enhancing the maturation and function of newly-generated inner ear cells.

In this review, we describe the recent advances in biomaterial applications for inner ear cell regeneration. We summarize the biomaterials that have been used for inner ear regeneration thus far, describe the advantages and limitations of these materials, analyze the mechanisms through which materials with different physicochemical properties act on inner ear cell regeneration, and depict the current status of material selection based on their characteristics to achieve the reconstruction of auditory circuits.

## 2 Biomaterials applied to inner ear cell regeneration

### 2.1 Hydrogels

Hydrogels have been widely used in stem cell bioengineering research (Liu et al., 2018), and according to our statistics, hydrogels may also be the most widely used biomaterial for inner ear cell regeneration. As hydrogels have excellent biocompatibility, bioactivity, and tunable mechanical properties (Liu et al., 2020), they can simulate the three-dimensional (3D) environment of the extracellular matrix (ECM) in which cells survive *in vivo* and always act as scaffolds to support cell growth in *in vitro* cultures (Evans et al., 2006). Matrigel is a kind of hydrogel extracted from the basement membrane of Engelbreth-Holm-Swarm mouse sarcoma cells (Arnaoutova et al., 2012). In addition to the shared advantages of hydrogels, Matrigel contains ECM components such as collagen, laminin, and nestin, as well as chemical cues for the maintenance of cell survival, such as growth factors, which provide complex tissue microenvironments that are more similar to those *in vivo* (Arnaoutova et al., 2012). Sarah et al. reported that matrigel was essential for the establishment of mouse embryonic stem cell (ESC)-derived inner ear organoids (Hocevar et al., 2021). Matrigel has been shown to promote neurite extension, maintain the electrophysiological function of purified SGN (Yan et al., 2018), and preserve the structure of SGN explant (Sun et al., 2016) *in vitro*. Matrigel has also been used to promote HC regeneration from Lgr5+ progenitor cells (Xia et al., 2023). However, batch-to-batch differences in the composition of Matrigel should be considered (Broguiere et al., 2018), which would inevitably lead to differences in cell culture results. Moreover, the elasticity modulus provided by Matrigel is insufficient to provide adequate support for cells (Soofi et al., 2009). To address these problems, hydrogels with defined components and improved mechanical properties have been investigated for their biological effects on inner ear regeneration (Rajasingh et al., 2017; Pouraghaei et al., 2020; Shi et al., 2023). One study demonstrated that Matrigel mixed with a certain ratio of alginate induced better differentiation of human gingival mesenchymal stem cells into auditory progenitor cells than Matrigel alone (Pouraghaei et al., 2020). Moreover, alginate modified by the Arg-Gly-Asp (RGD) sequence, a common cell adhesion peptide mainly derived from fibronectin, could also provide bioactive sites for cell-hydrogel interaction (Chikar et al., 2012). A single-component hydrogel crosslinked by self-assembling peptide amphiphiles with an Ile-Lys-Val-Ala-Val (IKVAV) epitope, another signal peptide epitope from laminin, has also been shown to promote the differentiation of hESCs into otic neural progenitors, as well as cell survival and localization after transplantation (Rajasingh et al., 2017). Interestingly, bacterial cellulose can also be fabricated into hydrogels for the differentiation and functional maintenance of SGNs (Shi et al., 2023). However, even if bioactive sites can be added or modified artificially, these single-component hydrogels are difficult to provide a complex and anisotropic biological environment similar to that of basement membrane extracts, which is especially required for the establishment of organoids (Shi et al., 2023). Hydrogels could also be used as carriers for inner ear delivery (Zhong et al., 2016; Chang H. T. et al., 2020) and as coatings for other synthetic materials and implants to reduce cytotoxicity owing to their exceptional biocompatibility (Chen et al., 2017; Hu et al., 2022). Another advantage of hydrogels for *in vivo* inner ear regeneration is that they provide a stem cell niche for transplanted stem cells and limit their dispersion (Nayagam et al., 2012).

At present, commonly used hydrogels are temperature-sensitive, such as Matrigel (Arnaoutova et al., 2012), or are formed through a cross-linking agent, such as alginate (Pouraghaei et al., 2020). GelMA, a photopolymerized hydrogel, is often used as a micropatterned topographical hydrogel prepared by combining gelatin and methacrylic anhydride (MA; Yue et al., 2015). It can be crosslinked and solidified into a gel form using ultraviolet (UV) or visible light in the presence of photoinitiators, enabling the design and adjustment of micron-scale morphological features using photomask to guide the directional growth of axons of SGNs (Clarke et al., 2011; Tuft et al., 2014a,b; Xu et al., 2018). A previous study demonstrated that the combination of topological GelMA and chemical cues could better guide and promote the neurite extension of SGNs by coating laminin on the grooves to facilitate neurite growth while coating EphA4-Fc on the ridges to repel it (Truong et al., 2021). Study of neurite-directed regrowth is necessary to guide regenerated neurons to connect with HCs in an orderly manner so as to establish auditory circuits.

### 2.2 Conductive nanomaterials

As afferent auditory signals are transducted mechanoelectrically by cochlear HCs and then transmitted into the cochlear nucleus by SGNs, maturation of the electrophysiological functions of regenerated HCs and SGNs is paramount (Sun et al., 2018). The electroactivity of biomaterials has been applied to the regulation of cell fate determination, especially in regenerated excitable cells such as cardiomyocytes and neurons (Ning et al., 2018). The known conductive material graphene and its surface modification products have been reported to have broad applications in the behavioral regulation of neurons (Tu et al., 2013) and neural stem cells (Guo et al., 2021). Recent studies have shown that the combination of graphene oxide and hydrogels can promote the expansion of SGN growth cones and the outgrowth of neurites (Shi et al., 2023). Graphene substrates have also been revealed to promote the proliferation and HC differentiation of inner ear Lgr5+ progenitor cells, despite having no effect on cell viability (Ding et al., 2022). MXene, an emerging two-dimensional layered material with a structure similar to that of graphene, has been used in a wide range of applications since its discovery (Naguib et al., 2011) due to its excellent hydrophilicity and electrical conductivity (Naguib et al., 2021). MXene has recently been applied in the *in vitro* study of SGNs and HCs, and was shown to promote the elongation of SGN neurites while maintaining their electrophysiological properties (Zhang et al., 2022). Carbon nanotubes (CNTs), classical conductive material with high mechanical strength and high electrical conductivity, are generally categorized as single-walled carbon nanotubes (SWCNTs) and multi-walled carbon nanotubes (MWCNTs; Mattson et al., 2000). CNTs have been shown to lead to the acquisition of longer and more aligned neurites in SGNs *in vitro* (Hu et al., 2022). In an interesting study combining CNTs onto butterfly wings with surface topologies, SGNs were not only able to grow directionally but also gained more mature synaptic functions (Wei et al., 2021). Nevertheless, in a biocompatibility study of CNTs, SGNs did not grow better when platinum electrodes were coated with CNTs even though the number of SGNs decreased in the SWCNTs group (Giugliano et al., 2016). This proves that CNTs are not well biocompatible; therefore, most of the conductive materials mentioned above have been mixed with hydrogels to improve their biocompatibility when applied. However, all of the above studies were conducted *in vitro*, and the roles of these materials in *in vivo* applications requires further investigation.

Conductive nanomaterials have also been studied as coatings for CI electrodes to explore the effect of appropriate electrostimulation on the neurite regrowth of damaged or degenerated SGNs, thus eliminating the electrode-neuron gap. Polypyrrole (Ppy), another conductive material, has been used as an electrode coating to facilitate electrical stimulation of SGNs (Richardson et al., 2009). Ppy is an electroactive polymer that composed of pyrrole monomers linked by negatively-charged dopants and exhibits electrical conduction based on its redox state without reducing the resistivity (Wallace and Kane-Maguire, 2002). Furthermore, Ppy is biocompatible and can be coupled with other bioactive materials; therefore, it can be used as an excellent substrate for cell adhesion (Richardson et al., 2007).

### 2.3 Superparamagnetic iron oxide

Superparamagnetic iron oxide (SPIO) nanoparticles (NPs) have been extensively studied as nanoscale drug carriers for nerve growth factors (Yuan et al., 2019) and have been reported to promote neuronal regeneration themselves (Yuan et al., 2018). Recent studies have demonstrated that SPIO NPs could be endocytosed by SGNs to promote the development of growth cones and neurite extension of SGNs with or without a magnetic field (Hu et al., 2021). In a study by Ye et al., SPIO NPs, after being endocytosed by mesenchyme stem cells (MSCs), induced the migration and homing of MSCs toward mouse cochleae under an external magnetic field, either by intratympanic or tail vein administration (Ahn et al., 2021). The movement of SPIO NPs relies on the magnetic field, a relatively less traumatic operation, which undoubtedly provides novel options for the application of stem cells in the inner ear as well as the neurite growth direction of regenerated SGNs.

### 2.4 Photomodulation and photo-responsive materials

Compared to biomechanical cues, electrical stimulation and magnetic fields, the application of light is more attractive because of its easier operation, minimal risk of trauma, and low toxic side effects (Yuan et al., 2019). Photomodulation initially applied to the inner ear demonstrated that 808 nm wavelength light emitting diode (LED) irradiation on the tympanic membrane of mice was effective in reducing ouabain-induced SGN degeneration (Lee et al., 2016). Following a study of HC differentiation from mouse ESC-developed organoids, 630 nm LED irradiation facilitated the differentiation of HC-like cells (Chang S.-Y. et al., 2020). Additionally, some common biomaterials respond to light and contribute to neuroregeneration (Yuan et al., 2019; Zheng et al., 2022). For example, gold (Au) NPs altered the level of cellular permeability and ion transport through photothermal effects from localized surface plasmon resonance, which resulted in inducing neuronal proliferation and differentiation (Yuan et al., 2019). CNTs could also respond to light and generate ultrasound through the photoacoustic effect to stimulate neuronal growth (Zheng et al., 2022). Unfortunately, these photo-responsive materials have not yet been used for inner ear cell regeneration.

## 3 Mechanisms of biomaterials acting on inner ear cell regeneration

**Figure 1:**
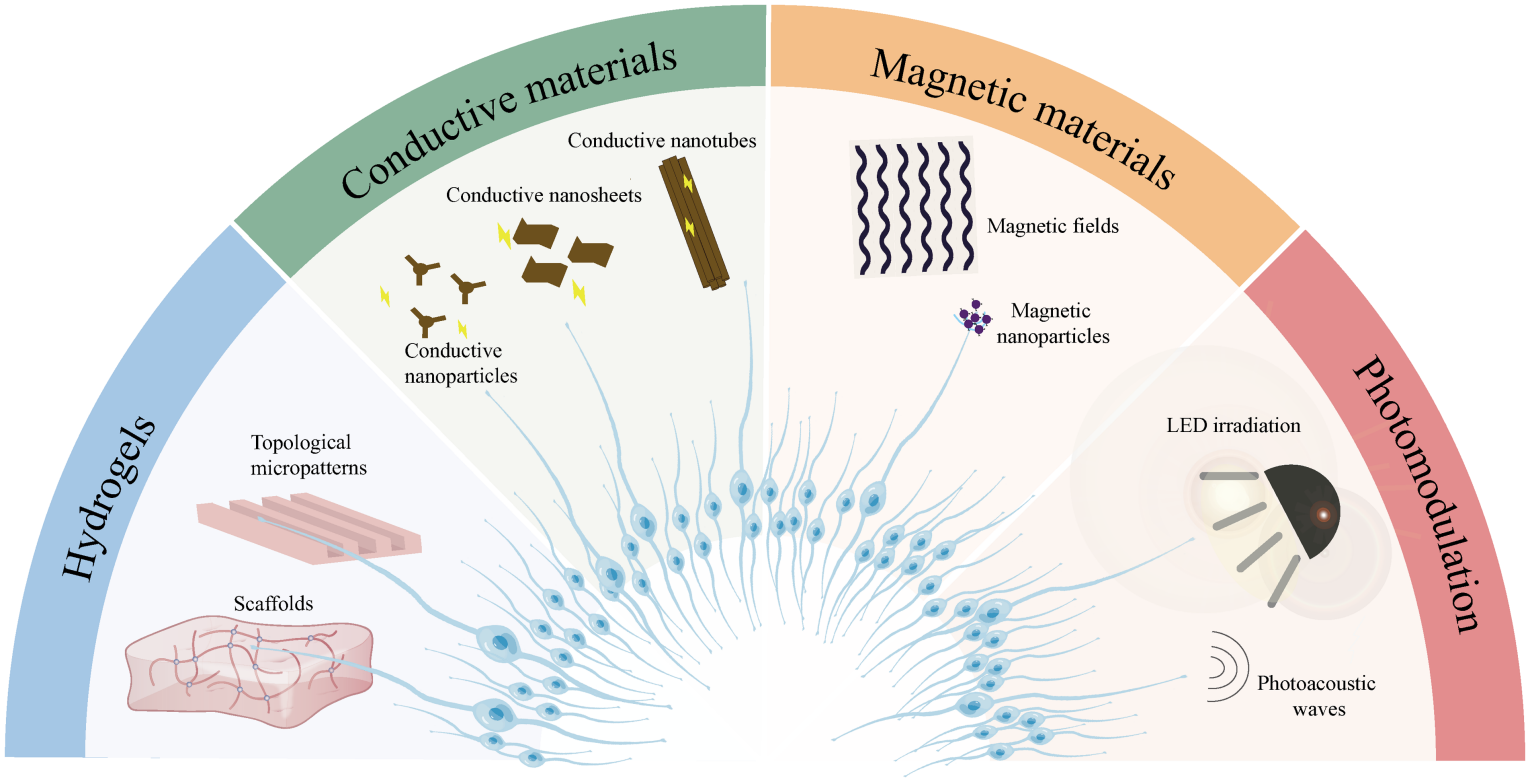
Schematic drawing of biomaterials used in the study of inner ear regeneration currently.

Almost all cells *in vivo* are housed in the ECM, which forms a complicated 3D microenvironment of fibers and interstitial spaces that provide abundant physical and biochemical cues and play an essential role in determining stem or progenitor cell fate and function (Arnaoutova et al., 2012). Therefore, it is crucial to select biomaterials with different properties to provide the most appropriate signals for the regeneration of cochlear cells (Figure 1). We analyzed the potential underlying mechanisms through which biomaterials may act on the inner ear regeneration process.

### 3.1 3D culture environment provided by biomaterial-based scaffolds

Scaffold-based 3D cell culture has now been applied in a broad range of fields, such as tissue engineering and regenerative medicine, and can simulate biophysical signals and biochemical cues provided by the ECM of the native microenvironment (Liu et al., 2018). Mechanical support, adhesion, and chemical gradients are key properties that are optimized in scaffold-based 3D culture systems and interact with each other to affect gene expression, cell signaling, and regulation of cell behavior. 3D culture systems are commonly created using hydrogels and are used for organoid establishment (Ishikawa et al., 2017). Composites of hydrogels and other scaffold materials have been reported to provide better mechanical support for the 3D culture of stem cells *in vitro* (Shi et al., 2023). The mechanical properties of the culture system, including bulk stiffness, local stiffness, and strain, have been considered extremely important for cell fate determination because mechanical stimuli can be converted into chemical signals that regulate gene expression (Bao et al., 2018; Jeon et al., 2021). The anisotropy of the structure and force direction provided by the scaffolds could influence cell morphology and even the expression of genes and proteins (Heo et al., 2011). For instance, when cultured in hydrogel-encapsulated scaffolds, nuclear localization of Yes-associated protein (YAP) was facilitated and the proliferation of the inner ear Lgr5+ progenitor cells was enhanced (Xia et al., 2020). In contrast, exposure to a conventional 2D culture plate, such as a stiff substrate of mouse embryonic fibroblasts, led to direct force transmission between the cytoskeleton and nucleus and disrupted the transport balance of the transcriptional regulator YAP in cells with flattened morphology (Elosegui-Artola et al., 2017).

The spatial distribution of adhesions around the cells, which link the intracellular cytoskeleton to the ECM, is also a prominent feature in the scaffold-based 3D culture system (Baker and Chen, 2012) and optimizes mechanotransduction from the ECM (Wang et al., 2009). Multidirectional ECM-cytoskeleton binding has been verified to be conducive to the formation of cellular actin-rich extension (Langevin et al., 2005), which may contribute to neurite growth. Hydrogels with modified cell adhesion-related signaling peptide epitopes, such as RGD (Chikar et al., 2012) or IKVAV (Frick et al., 2017; Rajasingh et al., 2017), have been reported to promote cell-hydrogel interactions and neuronal differentiation, which further demonstrated the importance of cell adhesion for inner ear cell regeneration.

A gradient of biochemical cues, such as exogenous or autocrine cytokines and growth factors, was formed in scaffold-based 3D cultures because the presence of scaffolds slowed down the diffusion of biochemical cues (Baker and Chen, 2012). Chemical cues with spatial gradients act effectively on target cells rather than those with a homogeneous distribution, which is conducive to the differentiation process of stem cells and critical for the maintenance of differentiated tissues (Liu et al., 2015). Furthermore, organoids cultured with scaffolds could receive anisotropic signaling cues relying on their various spatial locations within the aggregates, which often leads to the asynchronous differentiation of cells located at different depths and contributes to organoid establishment *in vitro* (Koehler and Hashino, 2014).

3D cultures with various scaffolds allow cells to self-organize freely and develop more naturally (Koehler and Hashino, 2014). Stem cells or inner ear progenitor cells cultured in 3D scaffolds can be induced to generate otic organoids, resulting in SGNs and HCs similar to those in native cochleae (Hocevar et al., 2021; Sun et al., 2023). Electrophysiological evaluations confirmed the identity and maturation of the newly generated HCs as well as the co-cultured SGNs (Xia et al., 2023). Moreover, functional synapses, which are remarkable biological structures that allow communication between neurons and their targets, were formed between SGNs and HCs (Xia et al., 2023). These studies suggested that the otic organoids formed *in vitro* could mimic the developmental pattern of cochleae *in vivo*. The establishment of functional inner ear organoids is of great significance to further investigate the molecular mechanisms involved in the development of the inner ear as well as facilitate studies on hearing disorders and drug screening (Koehler and Hashino, 2014; Munnamalai and Fekete, 2017).

### 3.2 Biomaterials-based coating for CI interfaces

CI technology has restored partial hearing in patients with SNHL over the past few decades; however, challenges still remain (Naples and Ruckenstein, 2020). Research on coating materials for CI electrodes has never stopped, and these coating materials, especially topologically structured (Clarke et al., 2011; Tuft et al., 2014a; Mattotti et al., 2015; Cai et al., 2016; Leigh et al., 2017; Xu et al., 2018; Truong et al., 2021) or conductive materials (Wei et al., 2021; Ding et al., 2022; Hu et al., 2022; Liao et al., 2022; Zhang et al., 2022), can be used as interfaces to promote axonal regrowth of degenerated SGNs.

The surface of GelMA can be designed by photomasking to form topological micropatterns with grooves and ridges, which guide the neurites to track the patterns closely and align strongly (Clarke et al., 2011). A study on neural stem cell found that GelMA with microgrooves may promote the directed extension of neurites by enhancing the expression of cell adhesion factors (Cai et al., 2022). Many other photopolymerized materials have been fabricated in the same way to control the spatial alignment of regenerated SGN neurites through surface topological patterns, which were expected to realize the precise connection of regenerated neurons to their targets (Tuft et al., 2014a; Leigh et al., 2017; Xu et al., 2018; Truong et al., 2021). Similarly, the topological microstructure of silicon micro-pillar substrates (Mattotti et al., 2015) and topographically modified nanocrystalline diamond (Cai et al., 2016) were also confirmed to play roles in guiding the orderly arrangement of SGN neurites while promoting their adhesion.

Electrical stimulation combined with conductive materials has also been shown to facilitate the extension of SGNs and promote the neuroelectric activity (Liao et al., 2022). Although the exact mechanisms through which electrical stimulation promotes neuronal regeneration are still unclear, the role of ion exchange, signaling pathways, and cytoskeletal rearrangements have been observed (Liu et al., 2021). First, ion exchange occurred in response to electrical stimulation modulates the membrane potential and neuronal electrical activity, such as calcium (Ca^2+^) transients (Cho et al., 1999; Wan et al., 2021). It is generally assumed that high-amplitude Ca^2+^ transients are generated by Ca^2+^ influx after the activation of voltage-gated Ca^2+^ channels (Murillo et al., 2017), and whereas low-amplitude Ca^2+^ transients are owing to the phospholipase C-mediated release of intracellular Ca^2+^ from endoplasmic reticulum (Khatib et al., 2004; Marino et al., 2015). In addition, the application of conductive materials could help transmit electrical signals more quickly and effectively, and the electrical signals could also be converted into biochemical cues to control cell behaviors (Thrivikraman et al., 2018). For example, the increase in intracellular Ca^2+^ concentration induced by electrical stimulation activates multiple signaling pathways, such as Ras/MAPK and PI3K/AKT, which regulate cell proliferation and differentiation (Zhao et al., 2020; Cheng et al., 2021). Membrane depolarization could also phosphorylate tyrosine in growth factor-like receptors and thus activate the cascade response of MAPK/ERK pathways (Sheikh et al., 2013). Furthermore, activated MAPK could trigger the c-Fos synthesis through the phosphorylation of cyclic AMP/Ca^2+^-responsive element-binding protein (Tan et al., 2007), and then regulate neuronal activity (Joo et al., 2015). For another reason, external electric fields may also affect cytoskeletal rearrangement by the reorganization of microtubes and actin filaments (Li and Kolega, 2002; Han et al., 2009; Thrivikraman et al., 2014), which are the main component of growth cones to direct the neurite outgrowth (Hur et al., 2011). Some mixtures of topological and conductive materials can simultaneously promote the functional recovery of SGNs through topological characterization and electrical stimulation (Wei et al., 2021).

As SGN degeneration occurs inevitably after HC damage (Kanzaki et al., 2002), an “electrode-neuron gap,” which exists between CI electrodes and the membranes at the terminal of SGNs neurites (Frick et al., 2017), and is usually produced after CI operation and considered to be the most remarkable obstacle in improving CI performance (Nella et al., 2022). The application of biomaterials can eliminate such “electrode-neuron gap” to a certain extent. For example, a resist pattern on the surface of nanocrystalline diamond has been designed to arrange specific neural interfaces, thereby creating independent electrical stimulation signals for individual neuron on the CI electrode array (Cai et al., 2016). Another research group managed to eliminate the “electrodes-neuron gap” by establishing a “neuro-regenerative nexus,” a concept that was first proposed in 2022 by Kevin et al. The “neuro-regenerative nexus” refers to the biological interface provided by transplanted hPSC-derived ONPs, the two neurites of which are expected to connect to native SGNs and CI electrodes, respectively (Nella et al., 2022). To provide more precise connections between the SGNs and the electrodes, researchers used PODS® crystals for continuous delivery of growth factors and hydrogel to provide a stem cell niche for ONPs after transplantation into the inner ear (Chang H. T. et al., 2020), and a device with microgrooves to control the gradient of growth factors (Nella et al., 2022). which research has contributed to the development of “biohybrid” CI.

### 3.3 Superparamagnetic iron oxide and magnetic fields

Magnetic NPs have been introduced to clinical applications for a long time, such as magnetic resonance imaging and their effects on biochemical processes have recently been studied. SPIO NPs, the only magnetic NPs used in inner ear regeneration studies, were found to be taken up by SGNs to promote their neurite extension (Hu et al., 2021). Two cellular uptake pathways of SPIO NPs have been identified, one of which was diffusion occurred at the NPs concentration of 10 μg/mL (Mustafa et al., 2011) and the other was endocytosis that occurred when nanoparticles were functionalized by NGF (Nam et al., 2009). SPIO NPs without MFs have also been reported to promote nerve growth, possibly through the release of Fe ions from SPIO NPs and their involvement in cell adhesion-mediated biological processes (Kim et al., 2011). The tensile force generated by endocytosed NPs driven by an external static MFs could stretch the ends of neurites and align them toward the direction of MFs (Riggio et al., 2014). SPIO NPs with dynamic MFs have also proved to promote neuronal differentiation and neurite orientation through mechanisms of the cytoskeletal forces (Yuan et al., 2018) and the increased intracellular Ca^2+^ concentration (Georgas et al., 2023).

### 3.4 Biochemical cues loaded-materials for inner ear regeneration *in vivo*

Neurotrophins have been shown to be essential for maintaining survival and promoting differentiation during regeneration of inner ear cells (Gillespie et al., 2003; Pettingill et al., 2008; Xia et al., 2023). However, owing to the unstable biochemical properties of neurotrophic proteins, their half-life is extremely short, leading to difficulties in the long-term regeneration studied *in vivo* after inner ear transplantation of stem cells (Glueckert et al., 2018). Therefore, growth factor-loaded biomaterials have been used in *in vivo* inner ear cell regeneration studies. For example, the aforementioned brain-derived neurotrophic factor (BDNF) loaded-PODS® crystals exhibited a slow, sustained, and stable release of BDNF after inner ear transplantation, maintaining a stable concentration of growth factors in the differentiation environment for stem cells *in vivo* (Chang H. T. et al., 2020). Ultra-high viscious alginate encapsulated with BDNF-overexpressing MSCs was reported to enable the continuous BDNF release and prevent the unpredictable migration of MSCs, which promotes the survival of rat SGNs and their neurite outgrowth (Schwieger et al., 2020). In another study, an anti-EGF antibody was loaded onto alginate microcapsules and implanted in the inner ear of guinea pigs, and EGF was consequently highly aggregated around the microcapsules and promoted microcapsule differentiation (Zhong et al., 2016). SPIO is also commonly used as a nanoscale drug carrier (Yuan et al., 2019), MSCs endocytosed with SPIO NPs could be homed into the inner ear by tympanic ventricular administration or caudal vein injection (Ahn et al., 2021), which illustrating the promising applications of that SPIO.

## 4 Conclusions and perspectives

Current studies on biomaterials with different properties applied to inner ear regeneration have focused on several broad directions such as serving as scaffolds to establish inner ear organoids, providing topological micropatterns to guide cell regrowth, enhancing electrical signals to facilitate the electrical activity of inner ear cells and the formation of neural networks, as well as loading with chemical cues to maintain the stable regeneration of stem cells *in vivo*. However, the present multiple approaches have not allowed for the functional maturity of regenerated cells or the establishment of auditory circuits. In order to achieve these ultimate goals, several issues must be addressed. First, the details of the application of biomaterials for inner ear regeneration need to be further confirmed, such as the mechanical properties of the scaffolds and the parameters of the electrical signals, because the extracellular environment should be tissue-specific. Second, most of the current studies have only includes the exploration of cellular behavior, with little research on the underlying mechanisms, which would be the foundation for the further development of material-based inner ear regeneration. Furthermore, due to the intricate extracellular environment and complicated process of cell fate determination, biomaterials should be strategically selected and combined in view of the obstacles to be solved in inner ear cell regeneration research.

## Author contributions

JL: Conceptualization, Writing – original draft, Data curation, Investigation. MW: Conceptualization, Writing – original draft, Data curation, Methodology. YM: Writing – original draft, Investigation. WA: Data curation, Writing – original draft. XW: Writing – original draft, Supervision. GS: Conceptualization, Investigation, Writing – original draft. HW: Funding acquisition, Writing – review & editing. WL: Conceptualization, Funding acquisition, Writing – review & editing, Supervision.

## Glossary

**Table 1:** 

SNHL	Sensorineural hearing loss
HCs	Hair cells
SGNs	Spiral ganglion neurons
CI	Cochlear implantation
DFAs	Diabetic foot ulcers
3D	Three-dimensional
ECM	Extracellular matrix
ESC	Embryonic stem cell
ONPs	Otic neural progenitors
MA	Methacrylic anhydride
CNTs	Carbon nanotubes
SWCNTs	Single-walled carbon nanotubes
MWCNTs	Multi-walled carbon nanotubes
Ppy	Polypyrrole
SPIO	Superparamagnetic iron oxide
NPs	Nanoparticles
NGFs	Nerve growth factors
MSCs	Mesenchyme stem cells
LED	Light emitting diode
LSPR	Localized surface plasmon resonance
YAP	Yes-associated protein
BDNF	Brain-derived neurotrophic factor
